# Membership of County Medical Societies

**Published:** 1885-08

**Authors:** 


### MEMBERSHIP OF COUNTY MEDICAL SOCIETIES.

In the New York County Medical Society, certain questions relating to membership were recently presented to its counsel, and, according to the *New York Medical Journal*, were answered as follows:

"1. Are all practitioners of medicine required by the laws of this State to become members of a county medical society? Answer—Yes.

2. How can membership in a county medical society be terminated? Answer—By the resignation of any member who shall also cease to practice medicine and surgery in the county.

3. If membership can be terminated by resignation, must the resignation be accepted by the society in order to make it operative? The reply was that a resignation was operative only in case the member ceased to practice his profession.

4. Is the statute of 1827, requiring practitioners of medicine to become members of a county society, still in force? Answer—It is, to the extent of requiring such membership.

5. Is the statute of 1841, requiring graduates from without the State to pass an examination by the Board of Censors of some county medical society in order to become legally qualified practitioners, still in force? Answer—It is not."

The reasons for these opinions were then given in full, and the report concluded with the following words: "Membership may be exacted of every practitioner, upon due notice, under penalty of forfeiture of the right to practice upon refusal. No active practitioner can resign from any county society, nor can the society accept the resignation of his membership."

According to this opinion, then, "quackery," "exclusive dogma," "unprofessional conduct," or anything else, cannot be raised by a county medical society of this State, as an excuse for refusing membership, discipline or expulsion. But our societies must submit to anything that is not actually criminal, without complaint and without means of redress. Since the laws of 1880 and subsequent amendments provide for the regulation of the practice of physic and surgery in ways independent of county medical societies, would it not be well for our law-making powers to ensure, in some manner, the means for a respectable membership, at least, in our county medical societies?

